# Klotho Polymorphism in Association With Serum Testosterone and Knee Strength in Women After Testosterone Administration

**DOI:** 10.3389/fphys.2022.844133

**Published:** 2022-05-03

**Authors:** Lena Ekström, Jona Elings Knutsson, Christina Stephanou, Angelica Lindén Hirschberg

**Affiliations:** ^1^ Department of Laboratory Medicine, Karolinska Institutet, Stockholm, Sweden; ^2^ Department of Women’s and Children’s Health, Karolinska Institutet, Stockholm, Sweden; ^3^ Department of Gynaecology and Reproductive Medicine, Karolinska University Hospital, Stockholm, Sweden

**Keywords:** testosterone, doping, AKR1C3, UGT2B17, SLCO2B1, knee strength, klotho

## Abstract

Administration of testosterone (T) is associated with increased serum T concentrations and improved physical performance in women. However, the inter-individual variation in T concentrations after T treatment is large and may in part be due to genetic variations. Serum T, as well as dihydrotestosterone (DHT), androstenedione (A) and the T/A ratio have been suggested as promising doping biomarkers for testosterone intake. Here, polymorphisms in androgen metabolic enzyme genes have been investigated in healthy women prior to and after 10 weeks administration of testosterone cream. Klotho is a protein that has been associated with anaerobic strength and here a genetic variation in klotho gene was studied in relation to performance as measured by isokinetic knee strength, as well as to serum androgen disposition. The AKR1C3 genotype (rs12529) was associated with serum T levels at baseline, whereas serum concentrations post T treatment did not differ between genotypes. The SLCO2B1 (rs12422149) and UGT2B17 deletion polymorphisms were not associated with serum concentration of either T, DHT or A. The klotho polymorphism (rs9536314) was associated with serum concentrations of both total T and T/A ratio after T administration. Individuals with the GT genotype increased T concentrations and T/A ratio more than women homozygous for the T allele. No significant difference in the association of klotho genotype with knee muscle strength was observed between placebo and T treatment. However, individuals homozygous for the T allele showed higher isometric mean torque scores at exit than GT subjects after T administration. This is the first time a genotype has been associated with androgen concentrations after T administration and muscle strength in women. Our results imply that subjects with a polymorphism in klotho may be more prone to detection using serum T and A as biomarkers.

## Introduction

Androgens are considered beneficial for athletic performance (Bhasin et al., 2001) (Eklund et al., 2017) and are therefore forbidden in sport. Administration of supra-physiological doses of testosterone (T) are known to increase muscle size and strength in men, (Bhasin et al., 1996) (Rogerson et al., 2007), and recently it was shown that 10 weeks transdermal T application in young, healthy women was associated with enhanced physical performance as assessed by running time to exhaustion whereas anaerobic physical performance was unchanged (Hirschberg et al., 2020).

It is possible that additional factors rather than a direct increase in T may contribute to anaerobic physical performance as assessed by e.g., knee strength. The klotho protein, often described as an “anti-age” protein has been associated with muscle strength in middle-aged and elderly populations (Koyama et al., 2015) (Amaro-Gahete et al., 2019). A functional T>G single nucleotide polymorphism (rs9536314) in the klotho gene has been associated with loss of enzymatic activity (Arking et al., 2002). It is possible that genetic variation in the klotho gene reflects anaerobic physical strength women, but this has not been studied before. Interestingly, klotho has been associated with T and dehydroepiandrosterone (DHEA) serum levels in healthy middle-aged adults (Dote-Montero et al., 2019) indicating a connection between androgens and klotho.

To test for doping with endogenous anabolic androgenic steroids (EAAS), urinary testosterone (T) and epitestosterone (E) are quantified, and a T/E ratio >4 is an indicative marker for T administration. Since 2016, the T/E ratio, as well as four additional urinary steroid ratios, are monitored in the athlete biological passport (ABP). The implementation of the ABP passport has considerably increased the chances to detect T doping both in men (Strahm et al., 2015) (Nair et al., 2020) and women (Elings Knutsson et al., 2021) (Salamin et al., 2021). Still some athletes may escape the detection radar, particularly women, and a future complementary testing approach may consider to also monitor serum steroids in an endocrine module of the ABP (Salamin et al., 2020) (Elings Knutsson et al., 2021). The androgens T, dihydrotestosterone (DHT), androstenedione (A) as well as T/A ratio have been suggested to function as biomarkers for testosterone intake (Salamin et al., 2021).

Notably, there is large inter-individual variability in serum T levels after transdermal administration of T in healthy males (Mullen et al., 2018) and females (Elings Knutsson et al., 2021). The reason behind the large differences in absolute serum concentrations and the fold increase post T administration may partly be due to genetic variations in genes coding for proteins/enzymes involved in steroidogenesis and androgen metabolism. Aldo-keto-reductase 1C3 (AKR1C3) is a promiscuous enzyme that participates in the biosynthesis and metabolism of a variety of substrates including androgens (Penning et al., 2000). A C>G polymorphism (rs12529) resulting in a histidine to glutamine change (H5Q) has been associated with cancer risk (Vaidyanathan et al., 2017), and men homozygous for the C-allele had higher serum T levels than G-carriers after T administration (Bhasin et al., 2018). Organic anion-transporting polypeptide (OATP) 1B1, an important drug transporter that mediates the hepatic uptake of many compounds, is encoded by the SLCO1B1 gene. The G>A polymorphism (s12422149), leading to amino acid change arginine to glutamine (R312Q), has been associated with higher T serum concentrations, both at baseline and after T administration in men (Schulze et al., 2012). Regrettably, neither the AKRIC3 nor SLCO2B1 SNPs have been studied in relation to T applications in women.

It is well known that the urinary steroid profile is highly influenced by a deletion polymorphism of the uridine diphospho-glucuronosyl transferase 2B17 (UGT2B17) gene, the main enzyme involved in T glucuronidation (Jakobsson et al., 2006). Individuals devoid of UGT2B17 are not reaching the population-based T/E cut-off of four after T administration (Schulze et al., 2008). However, the absence of UGT2B17 exerts no impact on circulatory T levels, neither at baseline nor after T administration in men (Ekstrom et al., 2011). But the UGT2B17 copy number variation (CNV) polymorphism has not been studied in relation to serum T levels in women administered with T.

The aim of this study was to study the association between AKR1C3 (rs12529), SLCO2B1 (rs12422149), and UGT2B17 deletion polymorphisms and the serum levels of T, DHT and A before and after 10 weeks daily T administration in women. Furthermore, to investigate the klotho polymorphism (rs9536314) in relation to steroid serum profile, as well as if physical performance as assessed by knee strength is dependent on the klotho genotype. Additionally, all polymorphisms were analyzed in relation to urinary steroid profile.

## Methods

### Study Population

The cohort includes 48 women from a randomized, double blind, placebo controlled, parallel study conducted at the Karolinska University Hospital, Stockholm, Sweden, between May 2017 and June 2018 (ClinicalTrials. gov ID: NCT03210558). Healthy women were recruited by advertisement, mainly from the Swedish School of Sports and Health Sciences. Inclusion criteria included: age 18–35 years, body mass index 19–25 kg/m^2^, non-smoking, a moderate to high self-reported level of recreational physical activity, not taking hormonal contraception and willing to use highly efficient non-hormonal contraception during the study period. In order not to infringe antidoping rules, the women had to agree not to participate in any sport competition event during the study period and for 1 month after termination of the study. Exclusion criteria were the presence of cardiovascular, liver, biliary or renal disease; hyperlipidaemia; uncontrolled high blood pressure; endocrinological disorders; oligomenorrhea or amenorrhea; pregnancy; history of thromboembolic disorder; any malignancy; and use of hormonal contraception in the 2 months prior to the study.

The study was approved by the regional ethics committee in Stockholm (2016/1485-32, amendment 2017/779–32) and was carried out in accordance with Good Clinical Practice and the World Medical Association Declaration of Helsinki—ethical principles for medical research involving human subjects. All women gave written informed consent. Participants were randomly assigned to treatment with placebo cream (1 ml) or testosterone cream 10 mg (1 ml) (AndroFeme 1) applied every evening to the upper outer thigh for 10 weeks. Demographic characteristics of the women are described in Table 1. A detailed description of study population has been described previously (Hirschberg et al., 2020).

**TABLE 1:** Clinical characteristics of the women in the testosterone group and the placebo group before and after treatment.

	Testosterone		Placebo	
	Before	After	Before	After
Age	28.4		28.4	
BMI kg/m^2^	23.3	23.4	23.0	23.2
Total lean mass g	47034	47773**	45418	45582
Testosterone nmol/L ±SD	0.9 (0.4)	4.3 (2.8)***	1.0 (0.4)	1.1 (0.4)

**p < 01, ***p < 0.001.

### Serum and Urinary Steroid Analyses and Physical Performance Analyses

The first baseline samples were all collected in the follicular phase of the menstrual cycle. Serum androgen concentrations (T, A and DHT) were quantified with LC-MS/MS method described in (Ke et al., 2014) and presented in our previous paper (Elings Knutsson et al., 2021). The urinary steroid profile including T, E, etiocholanolone (etio), androsterone (A), 5β-androstanediol (5βAdiol) and 5α-androstanediol (5αAdiol) were quantified with WADA accredited GC-MSMS method as described (Elings Knutsson et al., 2021). The physical performance tests presented in the original study (Hirschberg et al., - 2020) included knee extension torque.

### Genotyping

Blood samples from 46 women were available for DNA extraction (two samples missing), of which 23 were from women in the testosterone treatment group. DNA was extracted from 200 µL whole blood samples using PureLink^®^ Genomic DNA kit (Life Technology), and according to the manual. The DNA concentrations were determined on NanoDrop. Genotyping SNP analyses for AKRIC3 (rs12529), SLCO2B1 (rs12422149), and Klotho (rs9536314) were performed using following allelic discrimination assays; C___8723970_1_, C __3101331_10, C___2983037_20, all from Life Technology. The UGT2B17 deletion polymorphism the CNV assay ID Hs03285327_cn (Life Technology) was used. Also, the ubiquitously expressed RNaseP (assay ID 4403326, Life Technology) was used as an endogenous reference gene when calculating the presence of one or two UGT2B17 alleles with delta-delta CTT formula (Livak, 1999). Subjects with no UGT2B17 PCR signal but with RNaseP were identified as del/del. The final volume for the PCR reactions was 10 or 15 µL consisting of 20 ng DNA and 2xTaqman Fast Universal PCR Master Mix (ThermoFisher). The PCR was run on StepOne™ Real-Time PCR System with the fast program; 50°C 2 min, 95°C 20 s and 40 cycles of 95°C 3 s and 60°C 20 s. The fluorescence signals were analyzed with the StepOne software 2.3 (Applied Biosystems).

### Statistical Analyses

The isometric mean torque data were analyzed using a mixed model, with subjects as a random factor, and klotho genotype (GT, TT), treatment (testosterone and placebo), time (baseline and exit) and genotype*treatment, genotype*time, treatment*time and genotype*treatment*time as fixed factors. Genotype and treatment were the between group factors and time was the within group factor. The *p*-value for the interaction genotype*time was less than 0.10, and therefore we performed simple main effects tests, i.e. the effect of factor time was tested by holding the factor genotype fixed, or vice versa, averaged over the level of the factor treatment. The analyses were carried out with the statistical program R version 4.0.0 Copyright (C) 2020 The R Foundation for Statistical Computing.

Statistical analyses comparing steroid profile were performed using GraphPrism software version 8.3 from GraphPad (San Diego, CA, United States). For androgens showing normal distribution (T and A) the comparison between genotype groups was done with non-paired ANOVA (followed by Tukey`s test), or *t*-test. As DHT showed non Gaussian distribution, Kruskal Wallis (followed by Dunns multiple comparisons test) or Mann Whitney test were used. Differences were considered significant at the level *p* < 0.05 (2-sided test). One subject was homozygous for klotho GG genotype in the testosterone treatment group and was excluded from statistical analyses except for serum baseline levels where placebo and T group were pooled.

## Results

### Genetic Variation and Androgen Serum Profile Before and After Testosterone Administration

All genotypes distribution was in Hardy-Weinberg HW equilibrium.

The AKR1C3 polymorphism had an impact on serum T levels at baseline, individuals homozygous for the G-allele showed 35% higher serum levels than heterozygotes, *p* = 0.03 (Table 2). No association between the SLCO2B1, UGT2B17 and klotho deletion polymorphism and serum levels of any of the steroids were seen at baseline (Table 2).

**TABLE 2:** Distribution of polymorphisms in steroidogenic genes and androgen serum concentrations prior to intervention (at baseline) in the combined groups of women in the testosterone group and the placebo group (mean ± SD). Samples were taken in the follicular phase of the menstrual cycle (*n* = 44 women).

**AKR1C3 (rs12529)**	**CC (** ***n*** **= 8)**	**CG (** ***n*** **= 21)**	**GG (** ***n*** **= 17)**
Testosterone nmol/L ±SD	0.89 ± 0.36	0.81 ± 0.27	1.10 ± 0.38*
Dihydrotestosterone nmol/L ±SD	0.39 ± 0.15	0.37 ± 0.16	0.45 ± 0.17
Androstenedione nmol/L ±SD	4.37 ± 0.98	3.50 ± 0.72	4.51 ± 0.81
**SLCO2B1(rs12422149)**	**GG (** ***n*** **= 12)**	**AG (** ***n*** **= 34)**	
Testosterone nmol/L ±SD	0.94 ± 0.37	0.91 ± 0.30	
Dihydrotestosterone nmol/L ±SD	0.41 ± 0.18	0.37 ± 0.13	
Androstenedione nmol/L ±SD	4.16 ± 0.87	3.75 ± 0.75	
**UGT2B17 deletion**	**Ins/ins (** ***n*** **= 21)**	**Ins/del (** ***n*** **= 19)**	**Del/del (** ***n*** **= 6)**
Testosterone nmol/L ±SD	0.94 ± 0.53	0.96 ± 1.03	0.89 ± 0.38
Dihydrotestosterone nmol/L ±SD	0.40 ± 0.16	0.41 ± 0.19	0.38 ± 0.13
Androstenedione nmol/L ±SD	4.07 ± 0.76	3.97 ± 0.93	4.08 ± 0.91
**Klotho (rs9536314)**	**GG (** ***n*** **= 2)**	**GT (** ***n*** **= 11)**	**TT (** ***n*** **= 33)**
Testosterone nmol/L ±SD	1.30 ± 0.23	1.00 ± 0.47	0.88 ± 0.30
Dihydrotestosterone nmol/L ±SD	0.44 + 0.21	0.43 ± 0.21	0.39 ± 0.15
Androstenedione nmol/L ±SD	5.86 + 0.26	4.07 ± 0.76	3.91 ± 0.85

For the AKRIC3, SLCO2B1 and UGT2B17 genotypes, no differences in T, A and DHT absolute levels after 10 weeks topical testosterone administration were discerned. Moreover, the fold increase in the androgen concentrations observed after testosterone treatment did not associate with any of these genotypes (data not shown).

**FIGURE 1:**
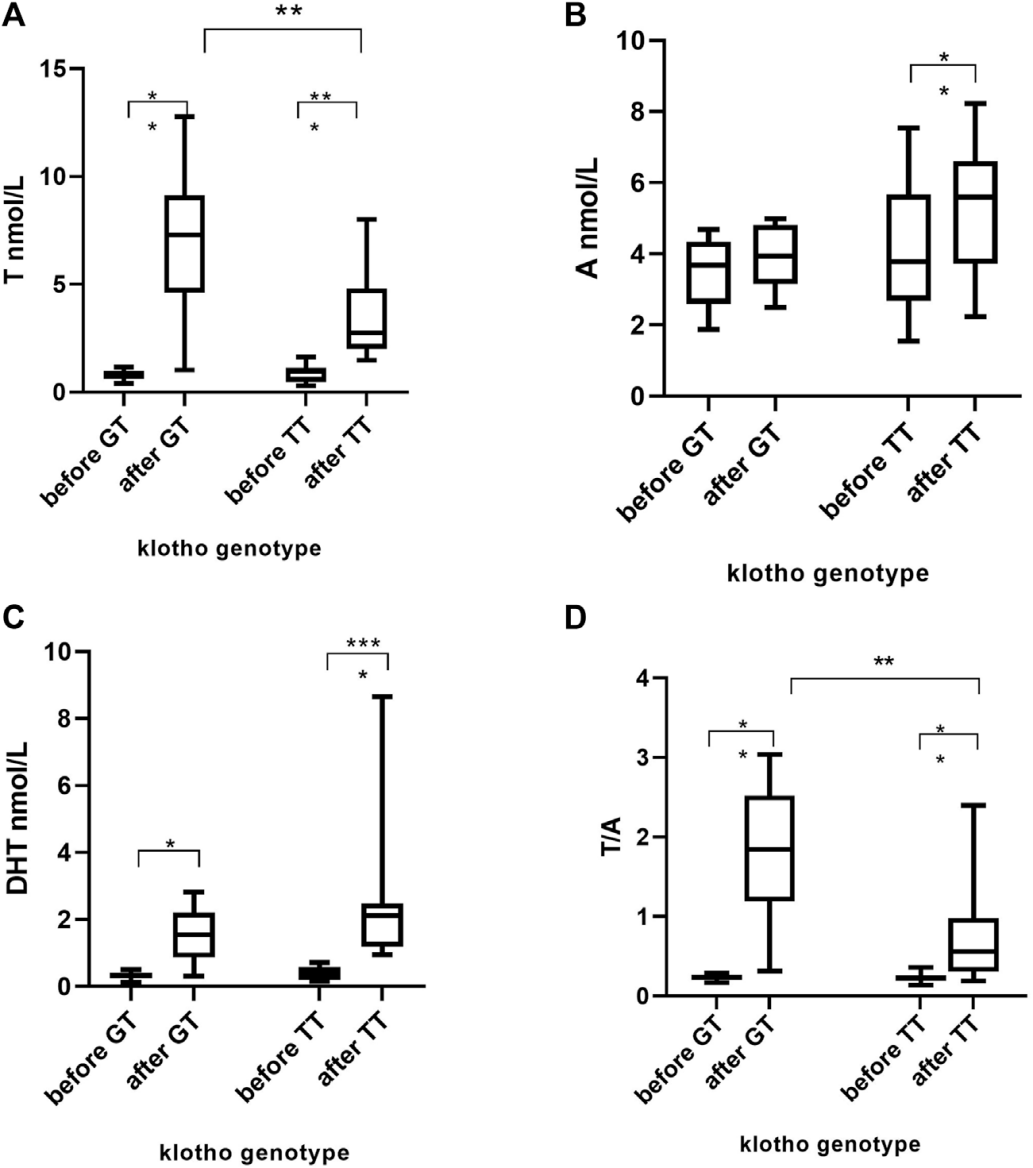
Serum concentrations of T, A, DHT and T/A before and after 10 weeks daily administration of T in 22 women. T = testosterone, A = androstenedione, DHT = dihydrotestosterone. *p < 0.05, **p < 0.01, ***p < 0.001, ****p < 0.0001.

The Klotho (rs9536314) genotype, however, was associated with T levels after the T treatment period, being 100% higher in heterozygous individuals as compared to individuals homozygous for the T allele, *p* = 0.009 (Figure 1A). The fold change in serum T was significantly associated with klotho genotype; women with two T alleles showed a mean T increase of 4.7-fold (SD ± 3.2) compared to G-allele carriers where a mean increase of 9.4-fold (SD ± 5.9) was noted, (*p* = 0.02). The A and DHT serum concentrations were not significantly associated with klotho genotype (Figures 1B,C).

The serum biomarker T/A did not differ between klotho genotype groups at baseline, Figure 1D. The mean T/A values in the testosterone group was 0.23 (range 0.19–0.36) priori to T administration. After 10 weeks of T administration, the mean T/A ratio was 140% higher in GT individuals as compared to TT subjects, *p* = 0.004 (Figure 1D). Subsequently, the fold increase in T/A was significantly higher in the heterozygotic participants, mean 7.5-fold (range 1.8–12.2), than TT mean 3.4-fold (range 0.8–9.1) *p* = 0.01.

As expected, no association between klotho genotype and serum steroid levels in the placebo group were found (data not shown).

### Genetic Variation and Androgen Urinary Steroid Profile Before and After Testosterone Administration

The UGT2B17 copy number variation polymorphism had a large impact on the urinary T and 5βAdiol levels. At baseline, individuals expressing UGT2B17 (ins/ins and ins/del) showed higher T (median 10.7 ng/ml and 7.6 ng/ml) and 5βAdiol (median 157 ng/ml and 143 ng/ml) concentrations compared to individuals devoid of UGT2B17 gene (0.61 ng/ml and 26 ng/ml for T and 5βAdiol, respectively), *p* < 0.001. After the T treatment period, the same pattern remained, individuals homozygous for the deletion allele showed lower T and 5βAdiol. Subsequently the increase in T/E as well as ABP ratios including T and 5βAdiol were associated with the UGT2B17 deletion polymorphism. Individuals homozygous for the klotho rs9536314 T-allele showed significantly lower T/E and higher A/T ratios than the heterozygotes subjects after T administration. The SNPs investigated in SLCO2B1 and AKR1C3 did not influence the urinary steroid profile. Supplementary File S1 shows the results for all urinary steroid metabolites and ABP-ratios in relation to different genotypes.

### Klotho SNP and Physical Performance

Since previous studies have found an association between klotho and muscle strength we investigated if the klotho SNP (rs9536314) was associated with isokinetic knee extension mean torque.

**FIGURE 2:**
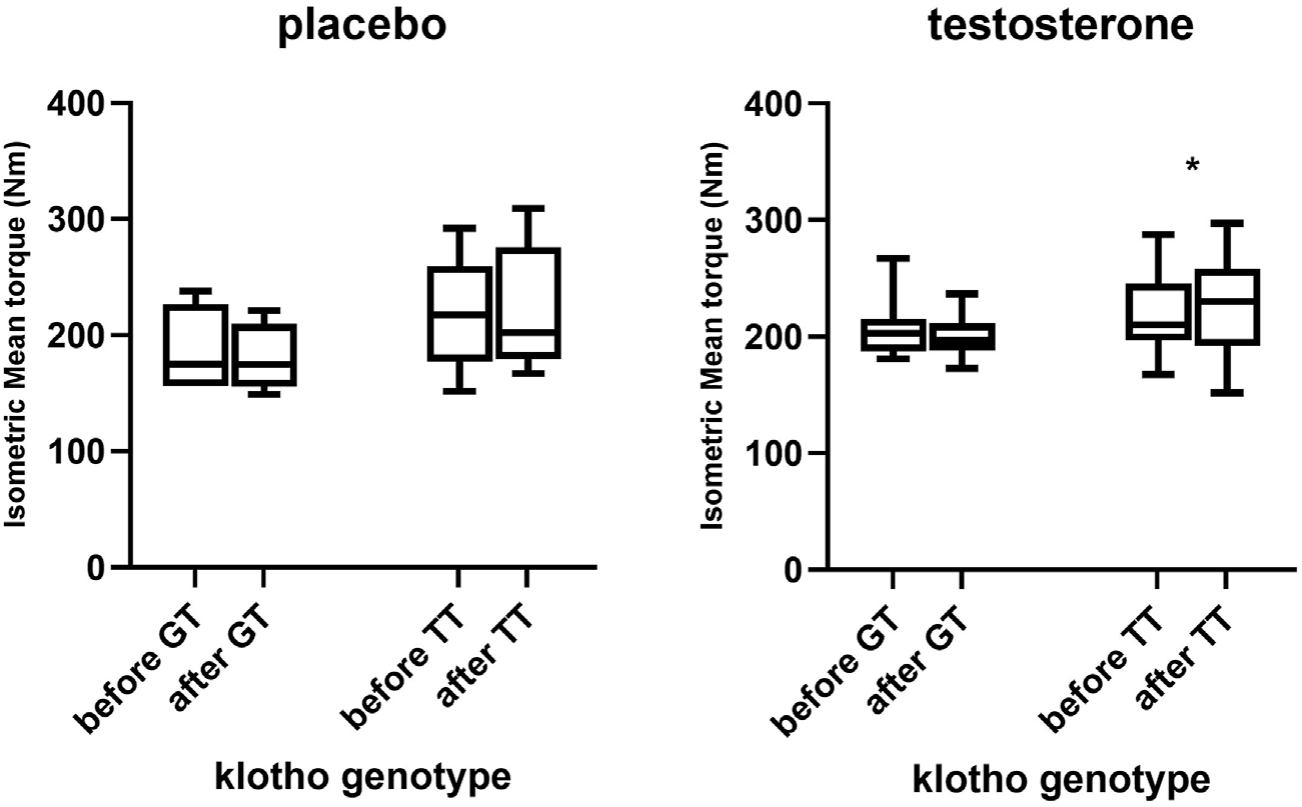
Percentage change in knee extension mean torque and peak torque in different klotho genotype groups in participants treated with testosterone and placebo respectively (*n* = 22 in each group). *p < 0.05.

There was no significant difference in mean torque between baseline and exit using treatment-genotype three factor interaction (*p* = 0.57). Furthermore, no significant interaction of klotho genotype*time was observed, even though a trend towards higher knee extension mean torque scores among TT subjects were discerned (*p* = 0.08). At exit, there was a significant difference in knee extension mean torque between TT and GT in the T group (*p* = 0.02), Figure 2.

## Discussion

This is the first time a genetic variation has been associated with serum T concentrations after T administration in healthy women. Individuals carrying one G-allele of the klotho SNP (rs9536314) had higher total testosterone serum levels after 10 weeks daily transdermal application of testosterone than homozygous for the more common T allele. However, at baseline and in the placebo group, there was no association between circulatory T and the klotho genotype suggesting different response of exogenous androgen between the genotype variants. Serum T are of interest to monitor in anti-doping testing, together with A and DHT as a complement to the urinary steroid profile. In addition to T, A and DHT, the T/A ratio has been suggested as a longitudinally stable biomarker for testosterone intake in women not influenced by the menstrual cycle (Salamin et al., 2021). After T administration, Salamin et al. observed an increase in T/A (Salamin et al., 2021) that corroborates with T/A values in our study participants, both before and after T treatment. The T/A ratio post T treatment in our study and the fold increase in T/A were associated with the klotho genotype, i.e., women homozygous for the TT allele displayed a lower T/A ratio and a smaller increase than those with the GT allel. Even though all women, regardless of genotype, increased their T/A (except one), the result indicates that women with GT klotho genotype may be more prone for detection using serum T/A as a biomarker.

The klotho SNP results in an amino acid change (F352V), that has been found to reduce the secretion of klotho in HeLa cells (Arking et al., 2002). The SNP, to our knowledge, has not been studied in relation to klotho serum levels, but theoretically reduced secretion may lead to lower klotho serum levels in individuals carrying a G-allele levels. Higher klotho serum levels have been associated with smaller decline in knee strength in older adults (Semba et al., 2016) This is in line with our hypothesis i.e., women homozygous for the T allele displayed higher scores in isokinetic muscle strength after T administration than subjects with a G-allele. Even though no significant association with treatment (placebo/testosterone) was noted, the role of T treatment can not be ruled out as genotype differences was noted in the T group but not in the placebo group. Notably, the genotype group that performed best in the muscle strength test is the variant associated with a smaller increase in serum T levels, and hence enhance our previous finding that T increase per se in serum does not mediate an effect on anaerobic strength. However, the circulatory T levels does not necessary reflect the androgen load in the muscles (Schiffer et al., 2018), and it is possible that the intracrine androgen metabolism differs between the klotho genotype groups. Another potential mechanism of the role of klotho in muscle strength might be indirectly via insulin-like growth factor I (IGF-I) signaling (Semba et al., 2016), as higher endogenous IGF-I and in insulin-like growth factor binding protein 3 (IGFBP-3) levels have been associated with klotho and androgens, as well as improved isometric knee strength (Jurimae et al., 2010). Future studies are warranted to elucidate the klotho polymorphism’s influence on muscle strength in healthy women.

In addition to klotho, genetic variations in metabolic enzymes AKR1C3, SLCO2B1 and UGT2B17 were studied. The AKR1C3 C>G polymorphism (rs12529) resulting in a non-conservative amino acid change (H5Q) has been associated for the first time with circulatory T concentrations in women. The result is in line with a study where men homozygous for GG exhibit higher baseline testosterone levels than GC/CC subjects (Shiota et al., 2020). However, after 10 weeks T administration no differences in serum T levels between the AKR1C3 genotype groups were determined, which is opposite to findings in men where individuals homozygous for the AKR1C3 (rs12529) CC allele displayed higher serum T compared G-carriers after testosterone replacement therapy (Bhasin et al., 2018). The reason may be sex differences.

Organic anion-transporting polypeptides (OATPs), which are encoded by SLCO, are a superfamily of membrane transport molecules that mediate the cellular uptake of steroid conjugates (Kinzi et al., 2021). A testosterone administration study in healthy men revealed that individuals homozygous for the SLCO2B1 (rs12422149) G-allele showed lower levels of total T prior to and 2 days after T injection, as compared to individuals expressing the A-allele (Schulze et al., 2012). However, here no association between SLCO2B1 genotype and androgen concentrations before and after T administration could be detected. A recent study indicated that OATP1B3 may be the main uptake transporter of T glucuronide, (Li et al., 2020). It is therefore possible that the involvement of OATP2B1 in T transport might be minor and exert a smaller impact in women where lower T increase are achieved.

The UGT2B17 gene deletion polymorphism is very well known to play a crucial rule in the urinary concentrations of T (Schulze et al., 2008), and to exert no impact on circulatory T levels in men (Ekstrom et al., 2011). Thirteen percent of the participants were homozygous for the UGT2B17 deletion allele and hence the genotype frequency match the phenotype frequency (i.e., T/E < 0.2) in our previous study including the same study population. The results herein confirm that the UGT2B17 CNV is not associated with serum T levels also in women neither at baseline nor after T administration. The urinary steroid profile was as expected connected to the UGT2B17 deletion polymorphism. Both T and 5βAdiol were excreted at much lower concentrations in subjects homozygous for the deletion variant in agreement with a previous study conducted in females (Schulze et al., 2014). Also, genetic variation in the klotho gene were associated with different T/E and A/T ratios post T administration, which is in line with higher serum T observed in GT subjects. However, the data interpretation was disturbed by the influence of UGT2B17 polymorphism, as del/del subjects were only represented in the TT panel, and the study cohort is too small to further analyze the contribution of klotho. Nevertheless, any putative association between the klotho SNP and the urinary steroid profile can be considered small and not of relevance in anti-doping testing.

A limitation with the study is relatively few subjects for genotype association studies and minor associations may be missed and/or risk of randomly false positive associations. The connection between circulatory androgens and klotho needs to be verified in future studies. Another drawback is that only one blood sample was taken after the last testosterone administration, and the time post last administration could vary between 8 and 12 h. Moreover, the compliance to T treatment is not known as this was not controlled for throughout the study. However, the increase in serum T at the end of treatment support good compliance. A future study should be controlled in regard of administration and sampling time-points and preferably also include more collection time points for fully pharmacokinetic assessment.

In conclusion, this is the first time a genetic variation in klotho has been linked to serum concentrations of T and muscle strength after T administration in women. This might have implications on detection sensitivity using T (and/or T/A ratio) as biomarker in a serum steroid passport.

## Data Availability

The datasets presented in this study can be found in online repositories. The names of the repository/repositories and accession number(s) can be found below: https://www.ebi.ac.uk/eva/, PRJEB51000.

## References

[B1] Amaro-Gahete F. J. De-la-O A. Jurado-Fasoli L. Ruiz J. R. Castillo M. J. (2019). Association of Basal Metabolic Rate and Fuel Oxidation in Basal Conditions and during Exercise, with Plasma S-Klotho: the FIT-AGEING Study. Aging 11, 5319–5333. 10.18632/aging.102100 31390595 PMC6710061

[B2] Arking D. E. Krebsova A. Macek M. Macek. Arking A. Jr. Mian I. S. (2002). Association of Human Aging with a Functional Variant of Klotho. Proc. Natl. Acad. Sci. U.S.A. 99, 856–861. 10.1073/pnas.022484299 11792841 PMC117395

[B3] Bhasin S. Storer T. W. Berman N. Callegari C. Clevenger B. Phillips J. (1996). The Effects of Supraphysiologic Doses of Testosterone on Muscle Size and Strength in Normal Men. N. Engl. J. Med. 335, 1–7. 10.1056/nejm199607043350101 8637535

[B4] Bhasin S. Travison T. G. O'Brien L. MacKrell J. Krishnan V. Ouyang H. (2018). Contributors to the Substantial Variation in On-Treatment Testosterone Levels in Men Receiving Transdermal Testosterone Gels in Randomized Trials. Andrology 6, 151–157. 10.1111/andr.12428 28981994

[B5] Bhasin S. Woodhouse L. Casaburi R. Singh A. B. Bhasin D. Berman N. (2001). Testosterone Dose-Response Relationships in Healthy Young Men. Am. J. Physiology-Endocrinology Metabolism 281, E1172–E1181. 10.1152/ajpendo.2001.281.6.e1172 11701431

[B6] Dote-Montero M. Amaro-Gahete F. J. De-la-O A. Jurado-Fasoli L. Gutierrez A. Castillo M. J. (2019). Study of the Association of DHEAS, Testosterone and Cortisol with S-Klotho Plasma Levels in Healthy Sedentary Middle-Aged Adults. Exp. Gerontol. 121, 55–61. 10.1016/j.exger.2019.03.010 30928678

[B7] Eklund E. Berglund B. Labrie F. Carlström K. Ekström L. Hirschberg A. L. (2017). Serum Androgen Profile and Physical Performance in Women Olympic Athletes. Br. J. Sports Med. 51, 1301–1308. 10.1136/bjsports-2017-097582 28646101

[B8] Ekström L. Schulze J. J. Guillemette C. Belanger A. Rane A. (2011). Bioavailability of Testosterone Enanthate Dependent on Genetic Variation in the Phosphodiesterase 7B but Not on the Uridine 5′-Diphospho-Glucuronosyltransferase (UGT2B17) Gene. Pharmacogenet Genomics 21, 325–332. 10.1097/fpc.0b013e328344c5c6 21383644

[B9] Elings Knutsson J. Andersson A. Baekken L. V. Pohanka A. Ekström L. Hirschberg A. L. (2021). Disposition of Urinary and Serum Steroid Metabolites in Response to Testosterone Administration in Healthy Women. J. Clin. Endocrinol. Metab. 106, 697–707. 10.1210/clinem/dgaa904 33274381

[B10] Hirschberg A. L. Elings Knutsson J. Helge T. Godhe M. Ekblom M. Bermon S. (2020). Effects of Moderately Increased Testosterone Concentration on Physical Performance in Young Women: a Double Blind, Randomised, Placebo Controlled Study. Br. J. Sports Med. 54, 599–604. 10.1136/bjsports-2018-100525 31615775

[B11] Jakobsson J. Ekström L. Inotsume N. Garle M. Lorentzon M. Ohlsson C. (2006). Large Differences in Testosterone Excretion in Korean and Swedish Men Are Strongly Associated with a UDP-Glucuronosyl Transferase 2B17 Polymorphism. J. Clin. Endocrinol. Metabolism 91, 687–693. 10.1210/jc.2005-1643 16332934

[B12] Jürimäe T. Pääsuke M. Kums T. Gapeyeva H. Ereline J. Saar M. (2010). Relationships between Contraction Properties of Knee Extensor Muscles and Fasting IGF‐1 and Adipocytokines in Physically Active Postmenopausal Women. Clin. Physiology Funct. Imaging 30, 344–348. 10.1111/j.1475-097x.2010.00950.x 20633033

[B13] Ke Y. Bertin J. Gonthier R. Simard J.-N. Labrie F. (2014). A Sensitive, Simple and Robust LC-MS/MS Method for the Simultaneous Quantification of Seven Androgen- and Estrogen-Related Steroids in Postmenopausal Serum. J. Steroid Biochem. Mol. Biol. 144, 523–534. 10.1016/j.jsbmb.2014.08.015 25158021

[B14] Kinzi J. Grube M. Meyer Zu Schwabedissen H. E. (2021). OATP2B1 - the Underrated Member of the Organic Anion Transporting Polypeptide Family of Drug Transporters? Biochem. Pharmacol. 188, 114534. 10.1016/j.bcp.2021.114534 33794186

[B15] Koyama D. Sato Y. Aizawa M. Maki T. Kurosawa M. Kuro-o M. (2015). Soluble αKlotho as a Candidate for the Biomarker of Aging. Biochem. Biophysical Res. Commun. 467, 1019–1025. 10.1016/j.bbrc.2015.10.018 26462468

[B16] Li C. Y. Gupta A. Gáborik Z. Kis E. Prasad B. (2020). Organic Anion Transporting Polypeptide-Mediated Hepatic Uptake of Glucuronide Metabolites of Androgens. Mol. Pharmacol. 98, 234–242. 10.1124/mol.120.119891 32587096

[B17] Livak K. J. (1999). Allelic Discrimination Using Fluorogenic Probes and the 5′ Nuclease Assay. Genet. Anal. Biomol. Eng. 14, 143–149. 10.1016/s1050-3862(98)00019-9 10084106

[B18] Mullen J. Börjesson A. Hopcraft O. Schulze J. J. Ericsson M. Rane A. (2018). Sensitivity of Doping Biomarkers after Administration of a Single Dose Testosterone Gel. Drug Test. Anal. 10, 839–848. 10.1002/dta.2341 29150907

[B19] Nair V. S. Husk J. Miller G. D. van Eenoo P. Crouch A. Eichner D. (2020). Evaluation of Longitudinal Steroid Profiling with the Adams Adaptive Model for Detection of Transdermal, Intramuscular, and Subcutaneous Testosterone Administration. Drug Test. Anal. 12 (10), 1419–1431. 10.1002/dta.2885 32578357

[B20] Penning T. M. Burczynski M. E. Jez J. M. Hung C.-F. Lin H.-K. Ma H. (2000). Human 3α-Hydroxysteroid Dehydrogenase Isoforms (AKR1C1‒AKR1C4) of the Aldo-Keto Reductase Superfamily: Functional Plasticity and Tissue Distribution Reveals Roles in the Inactivation and Formation of Male and Female Sex Hormones. Biochem. J. 351, 67–77. 10.1042/0264-6021:3510067 10998348 PMC1221336

[B21] Rogerson S. Weatherby R. P. Deakin G. B. Meir R. A. Coutts R. A. Zhou S. (2007). The Effect of Short-Term Use of Testosterone Enanthate on Muscular Strength and Power in Healthy Young Men. J. Strength Cond. Res. 21, 354–361. 10.1519/r-18385.1 17530941

[B22] Salamin O. Nicoli R. Langer T. Boccard J. Grundisch C. S. Xu C. (2021). Longitudinal Evaluation of Multiple Biomarkers for the Detection of Testosterone Gel Administration in Women with Normal Menstrual Cycle. Drug Test. Anal. [Online ahead of print.] 10.1002/dta.3040 33817997

[B23] Salamin O. Ponzetto F. Cauderay M. Boccard J. Rudaz S. Saugy M. (2020). Development and Validation of an UHPLC-MS/MS Method for Extended Serum Steroid Profiling in Female Populations. Bioanalysis 12, 753–768. 10.4155/bio-2020-0046 32479744

[B24] Schiffer L. Arlt W. Storbeck K.-H. (2018). Intracrine Androgen Biosynthesis, Metabolism and Action Revisited. Mol. Cell. Endocrinol. 465, 4–26. 10.1016/j.mce.2017.08.016 28865807 PMC6565845

[B25] Schulze J. J. Lundmark J. Garle M. Skilving I. Ekström L. Rane A. (2008). Doping Test Results Dependent on Genotype of Uridine Diphospho-Glucuronosyl Transferase 2B17, the Major Enzyme for Testosterone Glucuronidation. J. Clin. Endocrinol. Metab. 93, 2500–2506. 10.1210/jc.2008-0218 18334593

[B26] Schulze J. J. Mullen J. E. Berglund Lindgren E. Ericsson M. EkstrÃm L. Hirschberg A. L. n. (2014). The Impact of Genetics and Hormonal Contraceptives on the Steroid Profile in Female Athletes. Front. Endocrinol. 5, 50. 10.3389/fendo.2014.00050 PMC3989562 24782830

[B27] Schulze J. Johansson M. Rane A. Ekstrom L. (2012). Genetic Variation in SLCO2B1 Is Associated with Serum Levels of Testosterone and its Metabolites Prior to and Two Days after Testosterone Administration. Cppm 10, 226–230. 10.2174/187569212802510021

[B28] Semba R. D. Ferrucci L. Sun K. Simonsick E. Turner R. Miljkovic I. (2016). Low Plasma Klotho Concentrations and Decline of Knee Strength in Older Adults. Gerona 71, 103–108. 10.1093/gerona/glv077 PMC4706099 26359247

[B29] Shiota M. Endo S. Fujimoto N. Tsukahara S. Ushijima M. Kashiwagi E. (2020). Polymorphisms in Androgen Metabolism Genes with Serum Testosterone Levels and Prognosis in Androgen-Deprivation Therapy. Urol. Oncol. 38, e11–e18. 10.1016/j.urolonc.2020.06.033 32712140

[B30] Strahm E. Mullen J. E. Gårevik N. Ericsson M. Schulze J. J. Rane A. (2015). Dose-dependent Testosterone Sensitivity of the Steroidal Passport and GC-C-IRMS Analysis in Relation to the UGT2B17 Deletion Polymorphism. Drug Test. Anal. 7, 1063–1070. 10.1002/dta.1841 26198073

[B31] Vaidyanathan V. Naidu V. Kao C. H.-J. Karunasinghe N. Bishop K. S. Wang A. (2017). Environmental Factors and Risk of Aggressive Prostate Cancer Among a Population of New Zealand Men - a Genotypic Approach. Mol. Biosyst. 13, 681–698. 10.1039/c6mb00873a 28252132

