# Supplementary material for: Klotho Polymorphism in Association With Serum Testosterone and Knee Strength in Women After Testosterone Administration
Source: Front Physiol. 2022 May 3;13:844133. doi: 10.3389/fphys.2022.844133 (PMC9116293; doi:10.3389/fphys.2022.844133)
Supplement: Supplementary file 1 [file DataSheet2.PDF]

Supplementary file: Urinary concentrations of androgen metabolites and ABP ratios in relation to different genotypes at baseline and after ten weeks T treatment.

The data are presented as median and SD

baseline

ng/mL	AKR1C3 (rs12529)			p-value
	CC	CG	GG	
**Testosterone**	9.3 ± 6.4	8.4 ± 5.2	10.5 ± 6.6	0.7
**Androsterone**	2852 ± 924	3378 ± 1392	4046 ± 2138	0.48
**Epitestosterone**	10.6 ± 6.3	9.2 ± 3.7	14.7 ± 9.3	0.08
**Etiocholanolone**	3453 ± 799	3775 ± 1712	4089 ± 2040	0.9
**5β-androstanediol**	71.7 ± 29.7	147 ± 141	143 ± 128	0.2
**5α-androstanediol**	25.1 ± 6.6	35.5 ± 11.4	40.8 ± 27.1	0.1
**5α/5β**	0.38 ± 0.15	0.35 ± 0.2	0.48 ± 0.33	0.4
**T/E**	0.7 ± 0.39	0.95 ± 0.5	0.51 ± 0.42	**0.02**
**5α/E**	2.7 ± 0.7	4.1 ± 1.5	2.8 ± 1.1	**0.01**
**A/T**	2800 ± 6398	497 ± 293	1686 ± 1985	0.1
**A/Etio**	0.82 ± 0.2	0.93 ± 0.3	1 ± 0.3	0.3

after T treatment

AKR1C3 (rs12529)			p value
CC	CG	GG	
17.6 ± 16.4	23.6 ± 17	17.6 ± 8.7	0.9
3329 ± 1429	4093 ± 1339	3987 ± 2138	0.7
11.3 ± 5.1	12.6 ± 4.1	16 ± 8.2	0.5
3785 ± 1646	3383 ± 1944	3740 ± 1629	0.7
83.6 ± 54.2	176 ± 121	183 ± 140	0.3
44.5 ± 21.6	56.6 ± 30.7	57.7 ± 38.5	0.7
0.6 ± 0.25	0.49 ± 0.4	0.48 ± 0.36	0.5
1.93 ± 2.17	2 ± 1.34	1.07 ± 0.81	0.5
4.8 ± 3.12	4.6 ± 1.92	3.8 ± 2.21	0.6
1122 ± 1794	264 ± 180	650 ± 1249	0.7
0.88 ± 0.27	1.44 ± 0.52	1 ± 0.29	0.08

baseline

ng/mL	SLCO2B1 (rs12422149)		p-value
	GG	AG	
**Testosterone**	7.36 ± 5.87	9.9 ± 6.75	0.2
**Androsterone**	3635 ± 1774	3332 ± 1508	0.6
**Epitestosterone**	11.29 ± 7.75	12.42 ± 5.45	0.3
**Etiocholanolone**	3813 ± 1661	3930 ± 1969	0.8
**5β-androstanediol**	118.5 ± 104.4	173.5 ± 169.8	0.1
**5α-androstanediol**	37.25 ± 21	32.4 ± 12.7	0.6
**5α/5β**	0.44 ± 0.27	0.3 ± 0.22	0.1
**T/E**	0.73 ± 0.56	0.79 ± 0.35	0.4
**5α/E**	3.6 ± 1.47	2.92 ± 1.26	0.1
**A/T**	1653 ± 3253	442.8 ± 245.4	0.1
**A/Etio**	0.97 ± 0.31	0.88 ± 0.23	0.5

after T treatment

SLCO2B1 (rs12422149)		p value
GG	AG	
15.35 ± 12.57	22.43 ± 16	0.3
3891 ± 1873	3828 ± 1466	0.9
14 ± 7.45	13.59 ± 4.77	0.8
3451 ± 1764	4034 ± 1466	0.2
132.7 ± 125.8	213.3 ± 102.5	0.06
53.14 ± 31.94	57.15 ± 34.6	0.9
0.59 ± 0.35	0.34 ± 0.25	0.09
1.4 ± 1.33	1.9 ± 1.53	0.5
4.22 ± 2.11	4.58 ± 2.78	0.8
826.2 ± 1399	224.5 ± 134.2	0.2
1.22 ± 0.46	0.97 ± 0.28	0.4

baseline

ng/mL	UGT2B17 (CNV)			P-value
	**ins-ins**	**ins-del**	**del-del**	
**Testosterone**	10.69 ± 6.59	7.6 ± 4.36	0.61 ± 0.34	**0.0002**
**Androsterone**	3639.9 ± 1751.7	3634.2 ± 1833.1	3020.3 ± 1087.4	0.8
**Epitestosterone**	11.4 ± 6.13	11.86 ± 8.9	11.27 ± 5.26	0.9
**Etiocholanolone**	4064.4 ± 1734.3	3927 ± 1874	2870.6 ± 970.8	0.1
**5β-androstanediol**	157.4 ± 135.6	142.6 ± 121.4	26.75 ± 8.4	**0.0004**
**5α-androstanediol**	38.85 ± 20.36	36.98 ± 18.95	23 ± 11	0.09
**5α/5β**	0.3 ± 0.13	0.37 ± 0.24	0.83 ± 0.23	**0.0017**
**T/E**	1 ± 0.55	0.7 ± 0.26	0.05 ± 0.02	**0.0001**
**5α/E**	3.76 ± 1.58	3.47 ± 1.27	2.14 ± 0.52	**0.017**
**A/T**	445 ± 310	515.4 ± 177	6676.3 ± 5292.65	**0.0002**
**A/Etio**	0.89 ± 0.27	0.95 ± 0.29	1.07 ± 0.35	0.5

after T treatment

UGT2B17 (CNV)			p-value
**ins-ins**	**ins-del**	**del-del**	
15.91 ± 9.88	22.75 ± 15.3	3.23 ± 4.24	**0.0223**
4082.62 ± 2079.9	3973.3 ± 1664	2936 ± 698.5	0.6
16.78 ± 9.3	12 ± 4.18	12.81 ± 3.8	0.4
3737 ± 1737.5	3849 ± 1803.9	2591.66 ± 527.34	0.5
215.36 ± 119.74	151.32 ± 118.88	32.4 ± 4.37	**0.0175**
54.75 ± 36.65	60.07 ± 32.2	32.8 ± 2.97	0.5
0.32 ± 0.23	0.52 ± 0.32	1.01 ± 0.05	**0.016**
1.18 ± 1	2.17 ± 1.51	0.34 ± 0.51	**0.045**
3.69 ± 2.09	5.25 ± 2.38	2.69 ± 0.73	0.1
299.32 ± 130	255.71 ± 205.71	2919.2 ± 2301.1	0.09
1.16 ± 0.42	1.12 ± 0.49	1.12 ± 0.086	0.9

baseline

ng/mL	klotho (rs9536314		p-value
	**GT**	**TT**	
**Testosterone**	8.6 ± 5.9	7.4 ± 5.7	0.5
**Androsterone**	3919 ± 1796	3491 ± 1713	0.3
**Epitestosterone**	14.1 ± 11.9	10.7 ± 5.1	0.6
**Etiocholanolone**	3768 ± 1405	3740 ± 1807	0.8
**5β-androstanediol**	129.4 ± 116.9	132.6 ± 133.3	0.9
**5α-androstanediol**	41.2 ± 25.8	34.9 ± 17.1	0.8
**5αAdiol/5βAdiol**	0.43 ± 0.26	0.41 ± 0.26	0.6
**T/E**	0.73 ± 0.39	0.7 ± 0.42	0.7
**5αAdiol/E**	3.27 ± 1.09	3.52 ± 1.53	0.7
**A/T**	814.4 ± 1121	1551 ± 3230	0.4
**A/Etio**	1.04 ± 0.31	0.94 ± 0.26	0.2

after T treatment

klotho (rs9536314)		p value
**GT**	**TT**	
24.3 ± 14.9	15.2 ± 13.3	0.1
3665 ± 1502	4095 ±1797	0.9
10.5 ± 5	15.3 ± 7	0.1
3677 ± 1817	3611 ± 1722	0.9
128.4 ± 76.3	170.5 ± 141.2	0.8
54.8 ± 23.6	56.4 ± 35.4	0.6
0.47 ± 0.18	0.55 ± 0.39	0.8
2.81 ± 1.65	1.1 ± 0.98	**0.03**
5.79 ± 2.21	3.93 ± 2.11	0.09
194.3 ± 134.6	843.6 ± 1391	**0.04**
1.08 ± 0.48	1.21 ± 0.37	0.5
